# Supplementary material for: New Mitogenomes of the Polypedilum Generic Complex (Diptera: Chironomidae): Characterization and Phylogenetic Implications
Source: Insects. 2023 Feb 27;14(3):238. doi: 10.3390/insects14030238 (PMC10054901; doi:10.3390/insects14030238)
Supplement: Supplementary file 1 [file insects-14-00238-s001.zip › insects-2152035-supplementary.pdf]

Table S1. Sample resources are used in this study

| Speices | Sample ID | Topology | Accession Number | Reference |
|---|---|---|---|---|
| *Endochironomus albipennis* | XL1630 | Linear | OP950227 | This study |
| *Endochironomus pekanus* | XL2023 | Circle | OP950219 | This study |
| *Endochironomus tendens* | XL1631 | Linear | OP950228 | This study |
| *Phaenopsectra flavipes* | LGS279 | Circle | OP950216 | This study |
| *Polypedilum heberti* | XL821 | Linear | OP950225 | This study |
| *Polypedilum* sp.1 | NAM06 | Circle | OP950217 | This study |
| *Polypedilum nubifer* | / | Circle | MZ747090 | [1] |
| *Polypedilum vanderplanki* | / | Circle | KT251040 | [2] |
| *Polypedilum yongsanensis* | HHLY216 | Linear | OP950222 | This study |
| *Polypedilum unifascium* | / | Circle | MW677959 | [3] |
| *Polypedilum masudai* | XL2759 | Linear | OK513041 | This study |
| *Sergentia baueri* | XL2656 | Circle | OP950220 | This study |
| *Stictochironomus akizukii* | XL1229 | Circle | OP950218 | This study |
| *Stictochironomus juncaii* | XL1582 | Linear | OP950226 | This study |
| *Stictochironomus rosenschoeldi* | XL552 | Linear | OP950224 | This study |
| *Synendotendipes impar* | ITA1 | Linear | OP950223 | This study |
| *Synendotendipes* sp.1 | XL3845 | Circle | OP950221 | This study |
| *Stenochironomus baishanzuensis* | NLCH802 | Linear | OL742441 | [4] |
| *Stenochironomus okialbus* | ZJ761 | Circle | OL753645 | [4] |

**Reference**


1. Xiao, Y.L.; Xu, Z.G.; Wang, J.X.; Fang, X.L.; Fu, Y. Complete mitochondrial genome of a Eurytopic midge, *Polypedilum nubifer* (Diptera: Chironomidae). *Mitochondr. DNA Part B Resour.* **2022**, 7, 1936–1938. https://doi.org/10.1080/23802359.2022.2122746
2. Deviatiiarov, R.; Kikawada, T.; Gusev, O. The complete mitochondrial genome of an anhydrobiotic midge *Polypedilum vanderplanki* (Chironomidae, Diptera). *Mitochondrial DNA Part A* **2017**, *2*, 218–220. https:// doi.org/ 10.3109 /19401736.2015.1115849
3. Zheng, C.G.; Liu, Z.; Zhao, Y.M.; Wang, Y.; Bu, W.J.; Wang, X.H.; Lin, X.L. First report on mitochondrial gene rearrangement in non-biting midges, revealing a synapomorphy in *Stenochironomus* Kieffer (Diptera: Chironomidae). *Insects* **2022**, *13*, 115. https://doi.org/10.3390/insects13020115
4. Zheng, C.G.; Liu, Z.; Zhao, Y.M.; Wang, Y.; Bu, W.J.; Wang, X.H.; Lin, X.L. First report on mitochondrial gene rearrangement in non-biting midges, revealing a synapomorphy in Stenochironomus Kieffer (Diptera: Chironomidae). Insects 2022, 13, 115. https://doi.org/10.3390/insects13020115.

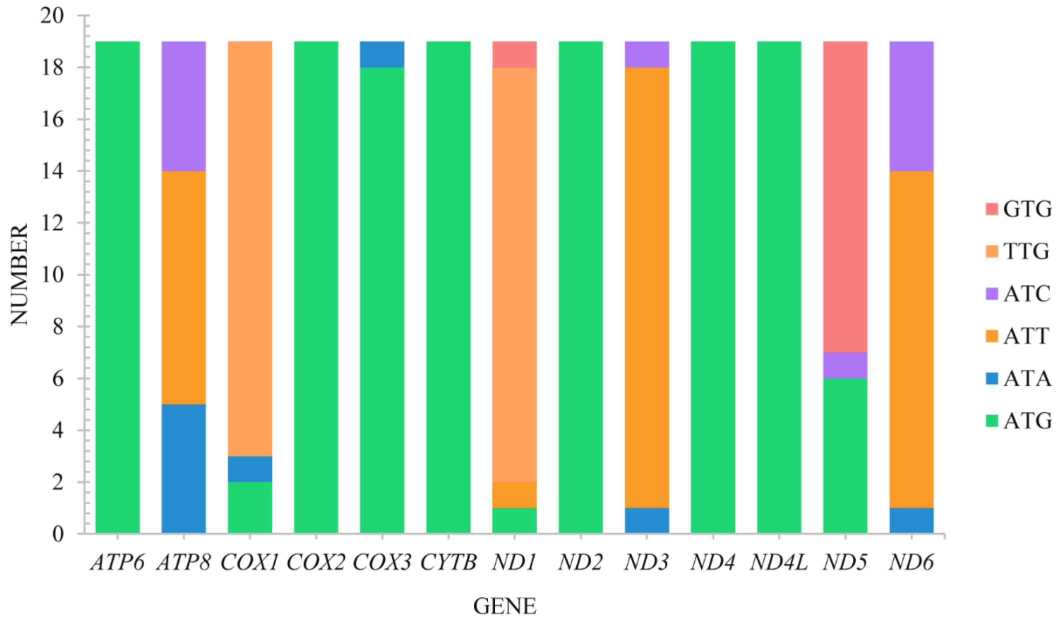


Figure S1. Start codons of protein-coding genes among *Polypedilum* generic complex mitogenomes

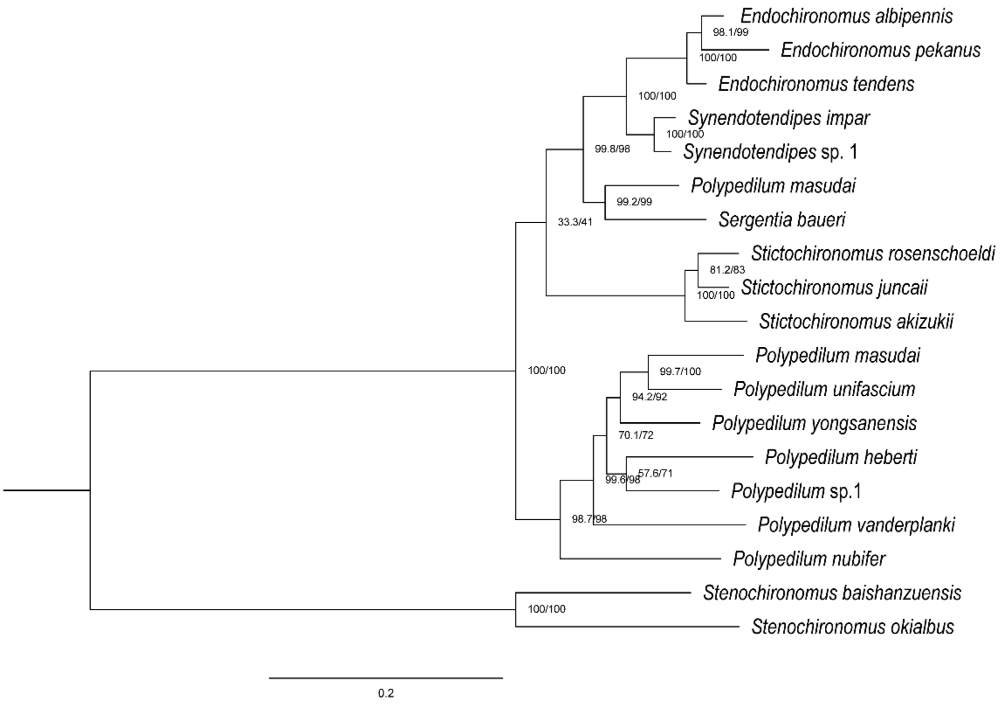


Figure S2. ML phylogenetic tree of *Polypedilum* generic complex based on the analysis cds_faa with Partition model in IQTREE. Support values on nodes indicate SH-aLRT/UFBoot2, respectively.

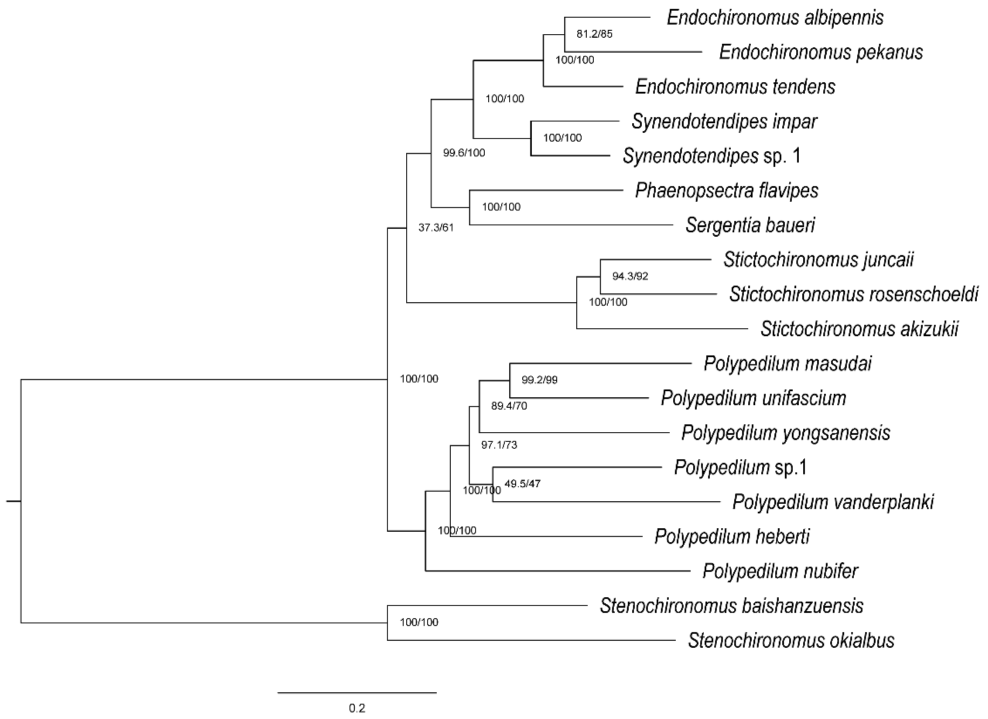


Figure S3. ML phylogenomic tree of *Polypedilum* generic complex based on the analysis cds_fna with Partition model in IQTREE. Support values on nodes indicate SH-aLRT/UFBoot2, respectively

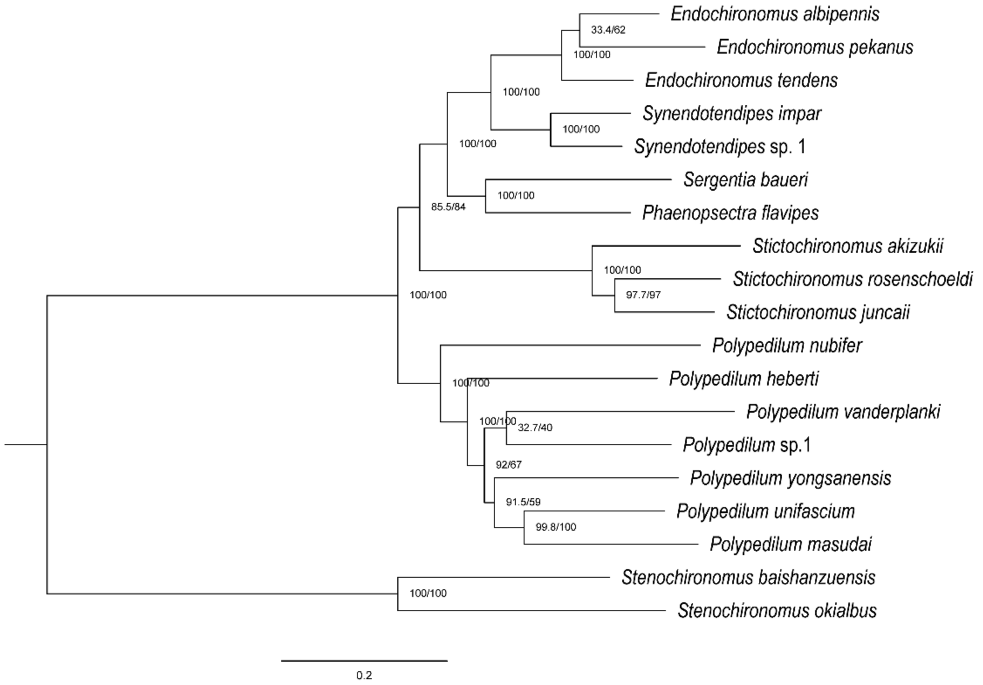


Figure S4. ML phylogenomic tree of *Polypedilum* generic complex based on the analysis cds_rrna with Partition model in IQTREE. Support values on nodes indicate SH-aLRT/UFBoot2, respectively

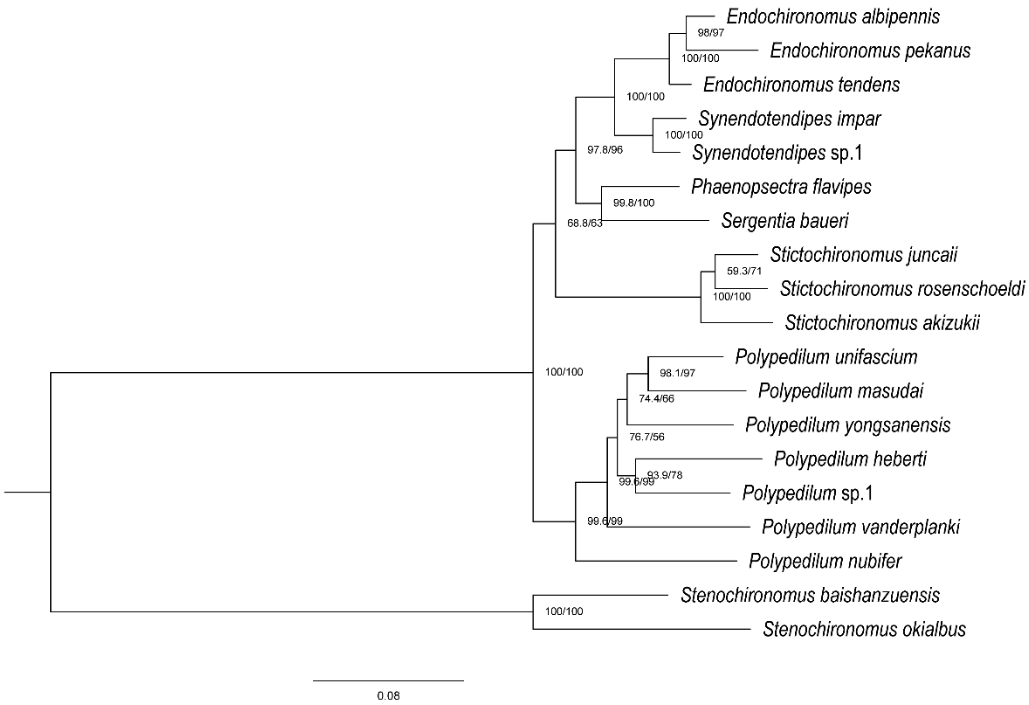


Figure S5. ML phylogenomic tree of *Polypedilum* generic complex based on the analysis cds12_fna with Partition model in IQTREE. Support values on nodes indicate SH-aLRT/UFBoot2, respectively

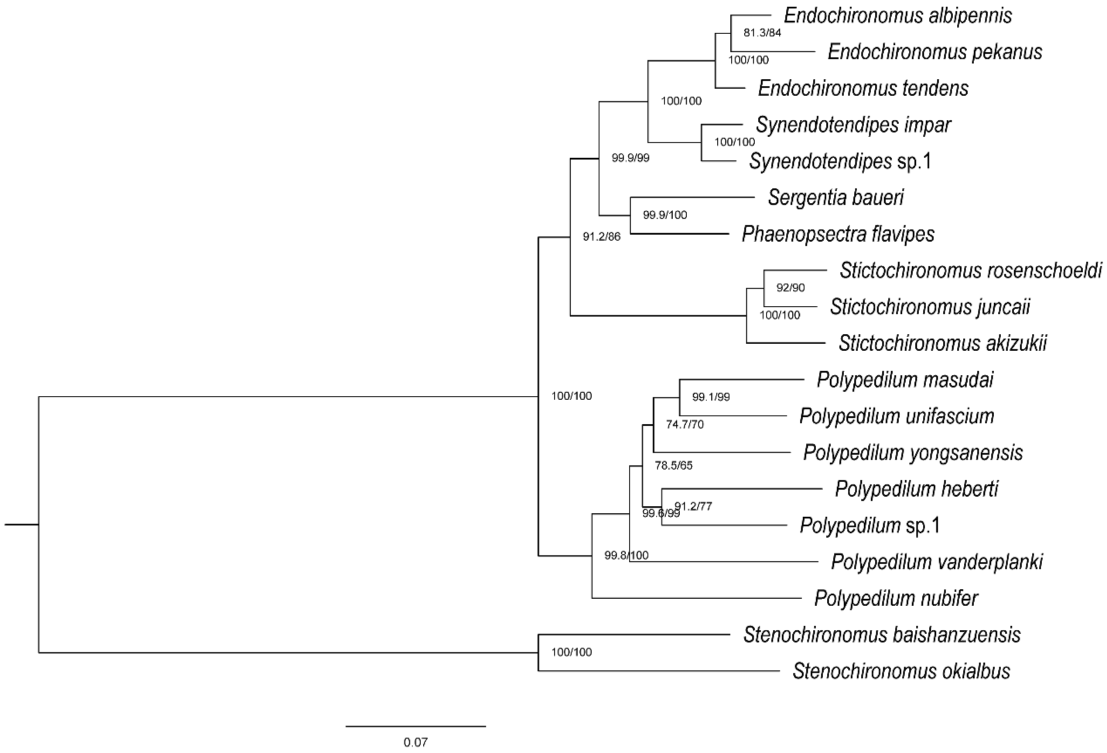


Figure S6. ML phylogenomic tree of *Polypedilum* generic complex based on the analysis cds12_rrna with Partition model in IQTREE. Support values on nodes indicate SH-aLRT/UFBoot2, respectively

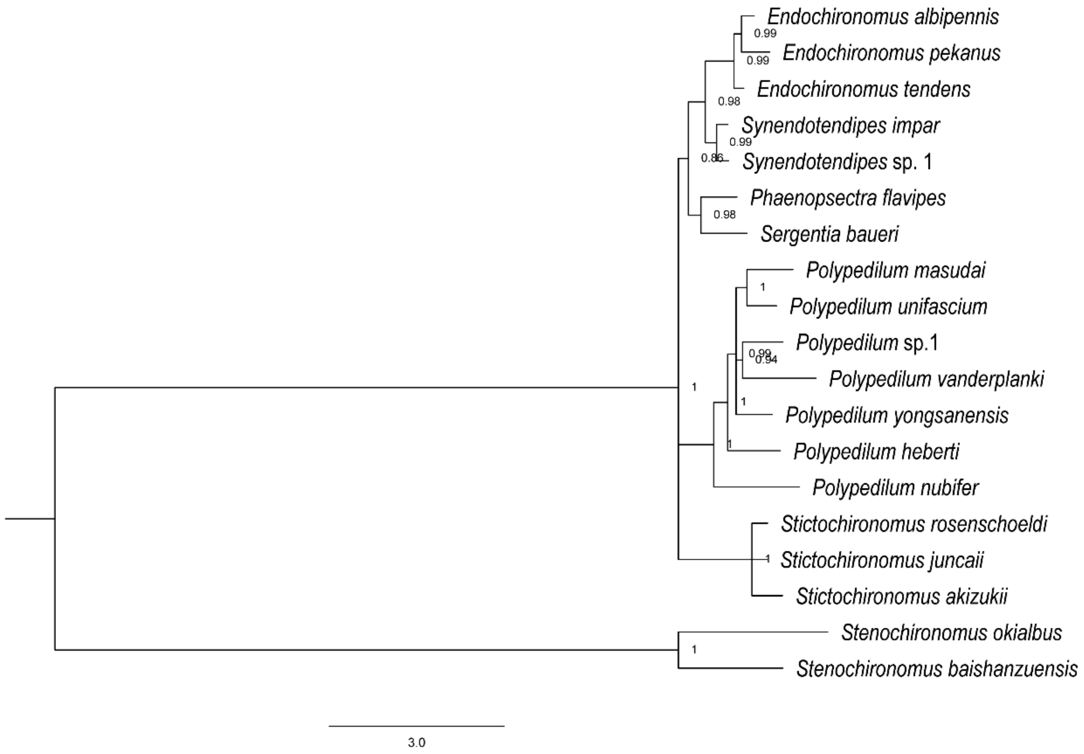


Figure S7. BI phylogenomic tree of *Polypedilum* generic complex based on the analysis cds_fna with GTR+CAT model in phylobayes. Support values on nodes indicate Bayesian posterior probabilities.

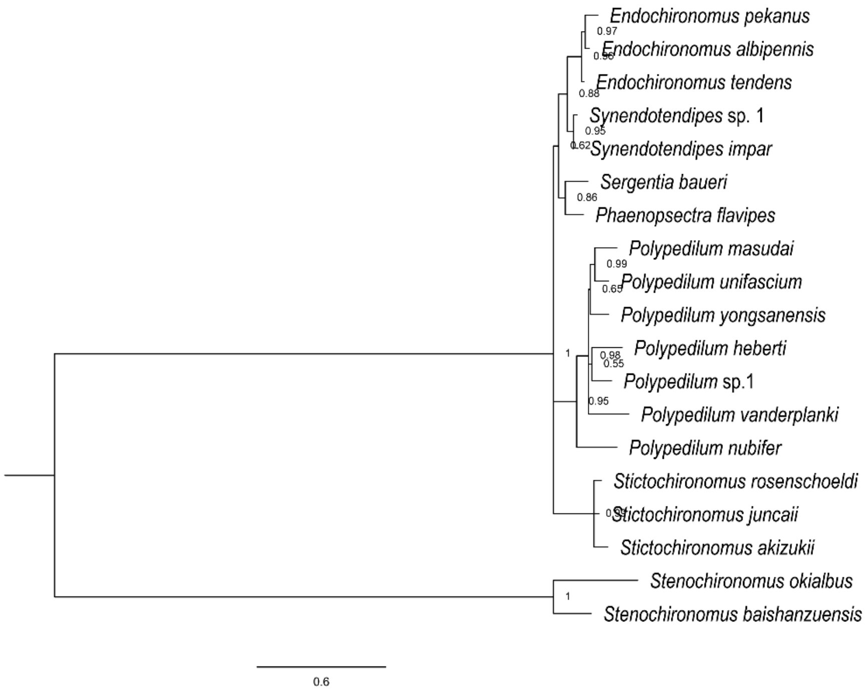


Figure S8. BI phylogenomic tree of *Polypedilum* generic complex based on the analysis cds12_fna with GTR+CAT model in phylobayes. Support values on nodes indicate Bayesian posterior probabilities.

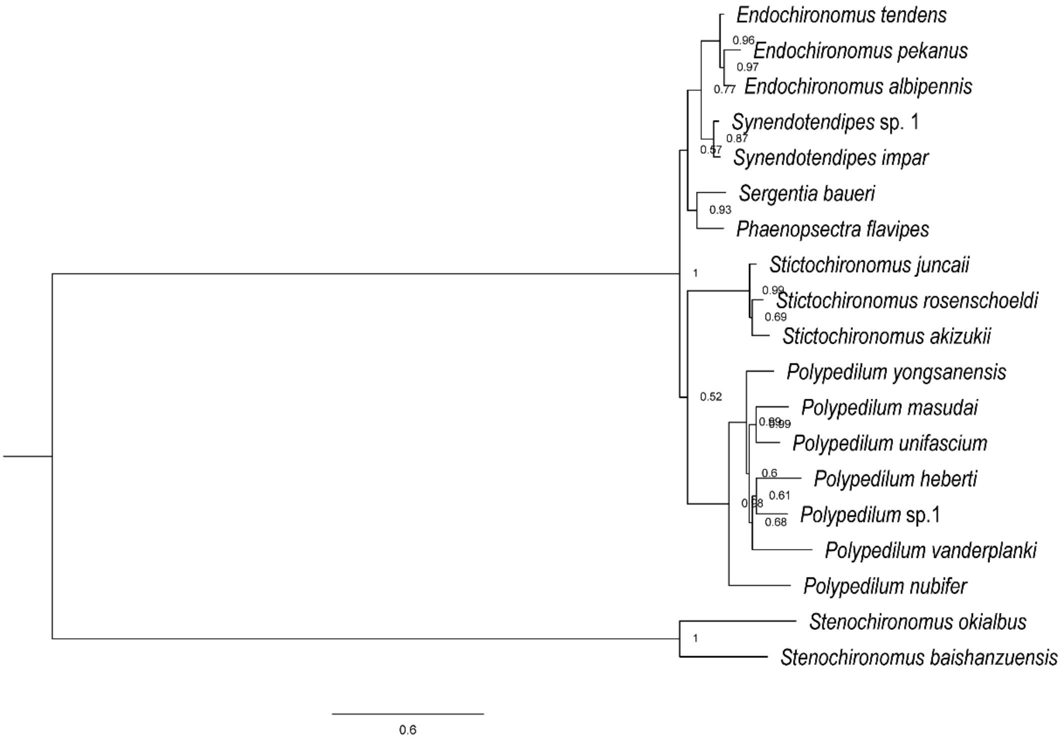


Figure S9. BI phylogenomic tree of *Polypedilum* generic complex based on the analysis cds12_rrna with GTR+CAT model in phylobayes. Support values on nodes indicate Bayesian posterior probabilities.
